# Subacute liver failure caused by immunotherapy in a patient with gastric cancer was improved after multimodal treatment with artificial liver combined with liver-protective drugs: a case report

**DOI:** 10.3389/fimmu.2026.1755661

**Published:** 2026-02-13

**Authors:** Yueer Wang, Yanfei Wen, Yuxin Jiang, Chen Li, Haiyan Liu

**Affiliations:** Department of Oncology, The Second Affiliated Hospital of Shandong First Medical University, Tai’an, Shandong, China

**Keywords:** artificial liver, case report, gastric cancer, immunotherapy, liver-protective drugs, multimodal treatment, subacute liver failure

## Abstract

The emergence of the anti-tumor immunotherapy era has led to the widespread adoption of immune checkpoint inhibitors (ICIs) in clinical practice. While ICIs exhibit substantial anti-tumor efficacy across a broad range of malignancies, they may also induce a diverse spectrum of immune-related adverse events (irAEs), involving multiple organ systems and, in severe instances, resulting in life-threatening complications. This report describes a rare case of severe immune-mediated hepatotoxicity (IMH) in a 76-year-old male patient with postoperative gastric cancer who was undergoing adjuvant therapy with camrelizumab in combination with oxaliplatin and S-1 (SOX regimen). The condition presented as CTCAE v5.0 Grade 4 IMH and subsequently progressed to subacute liver failure (SALF). Following an inadequate response to conventional hepatoprotective agents, glucocorticoids, and immunosuppressants, the patient received artificial liver support therapy consisting of two sessions of plasma exchange (PE) and three sessions of a double plasma molecular adsorption system (DPMAS), administered in an alternating sequence. After this multimodal intervention, liver function demonstrated marked recovery and returned to normal levels four months after discharge. No tumor recurrence occurred during follow-up, and hepatic function remained stable through August 2025. This case highlights the critical need for early recognition and prompt, comprehensive management of severe ICI-related liver injury and supports the potential clinical utility of artificial liver support systems in such scenarios.

## Introduction

Immune checkpoint inhibitors (ICIs) enhance the anti-tumor immune response by effectively relieving tumor-mediated immune suppression (1). Camrelizumab is a humanized monoclonal antibody targeting programmed cell death protein 1 (PD-1) that has demonstrated notable anti-tumor activity and clinical efficacy in advanced hepatocellular carcinoma and gastrointestinal malignancies, with a favorable safety profile (2, 3). However, irAEs induced by ICIs may affect multiple organs and systems, resulting in varying degrees of tissue damage. Immune-mediated hepatotoxicity (IMH) is a distinct form of hepatotoxicity specifically associated with ICI therapy.

The diagnosis of IMH primarily relies on abnormal liver function tests, with exclusion of other potential etiologies of liver injury, such as active viral hepatitis, autoimmune hepatitis, and hepatic metastases. The severity of IMH is classified into grades 1 to 5 according to the CTCAE v5.0 (4). During IMH, ALT and/or AST are typically markedly elevated, with or without concomitant increases in bilirubin levels. Most cases lack specific clinical symptoms. Elevated bilirubin is often indicative of an increased risk of liver failure, warranting close clinical monitoring and timely implementation of more aggressive intervention strategies (5).

Currently, the management of IMH remains limited and primarily relies on glucocorticoids and certain immunosuppressive agents. According to the “Guidelines for the diagnosis and treatment of Drug-induced liver Injury (2023 Edition)” issued by the Chinese Society of Clinical Oncology (CSCO), foundational hepatoprotective therapies mainly encompass anti-inflammatory agents, liver cell membrane stabilizers, and drugs that promote biliary excretion (6). Given the complex and heterogeneous pathogenesis of IMH, combination pharmacotherapy is commonly employed in clinical practice based on individual patient profiles, aiming to achieve multi-target intervention and synergistic effects. The use of glucocorticoids should be tailored according to the severity of liver injury. For glucocorticoid-refractory cases, mycophenolate mofetil (MMF) has been established as an effective second-line therapeutic option; however, there remains a lack of consensus regarding optimal third-line treatment strategies (6).

The primary treatment modalities of non-biological artificial liver support systems include plasma exchange (PE), double plasma molecular adsorption system (DPMAS), and continuous renal replacement therapy (CRRT). Among these, PE is currently recognized as the most effective liver support therapy and is the only intervention explicitly recommended by existing clinical guidelines. The European Association for the Study of the Liver (EASL) Clinical Practice Guidelines on Acute Liver Failure and the American Association for the Study of Liver Diseases (AASLD) guidelines both list artificial liver support therapy as a key management strategy for acute liver failure, clearly indicating its clinical efficacy in specific patient populations (7). Based on several retrospective controlled studies by Zhou, Zhong, Guo et al. (8–10), it has been demonstrated that the efficacy of DPMAS combined with PE is comparable to that of full-volume PE in the treatment of liver failure, with potential to further improve short-term outcomes in patients with acute liver failure. When plasma supply is limited, therapeutic efficacy can be maintained even with reduced plasma dosage (11). This case reports a patient with severe IMH who received multimodal therapy comprising hepatoprotective agents, glucocorticoids, and immunosuppressants, in combination with an artificial liver support system, and achieved a favorable clinical outcome.

## Case presentation

### Chief complaint

A 76-year-old male presented to the Second Affiliated Hospital of Shandong First Medical University in July 2023 with a one-week history of fatigue, loss of appetite, and jaundice.

### History of present illness

**Figure 1:**
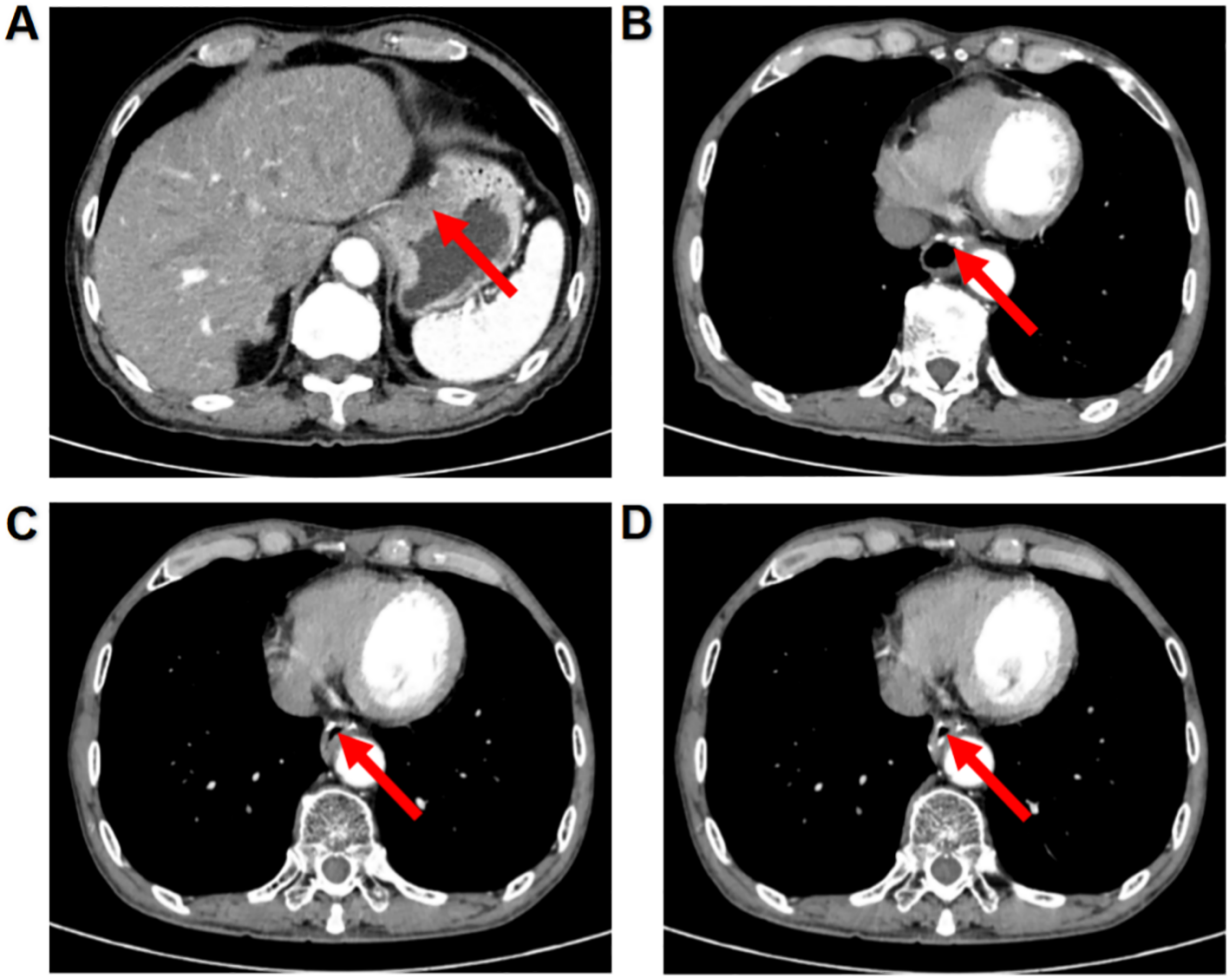
Imaging evaluation of the patient from diagnosis onward. **(A)** Abdominal contrast-enhanced CT image on March 30, 2023, revealing a mass located at the gastric fundus–lesser curvature junction. **(B)** Non-contrast abdominal CT scan on July 5, 2023, performed during the episode of immune-mediated subacute liver failure. **(C)** Contrast-enhanced abdominal CT on November 9, 2024, showing postoperative changes following resection of the gastric mass, with no evidence of recurrence and stable disease. **(D)** Contrast-enhanced abdominal CT on June 5, 2025, demonstrating continued stability without tumor recurrence.

**Figure 2:**
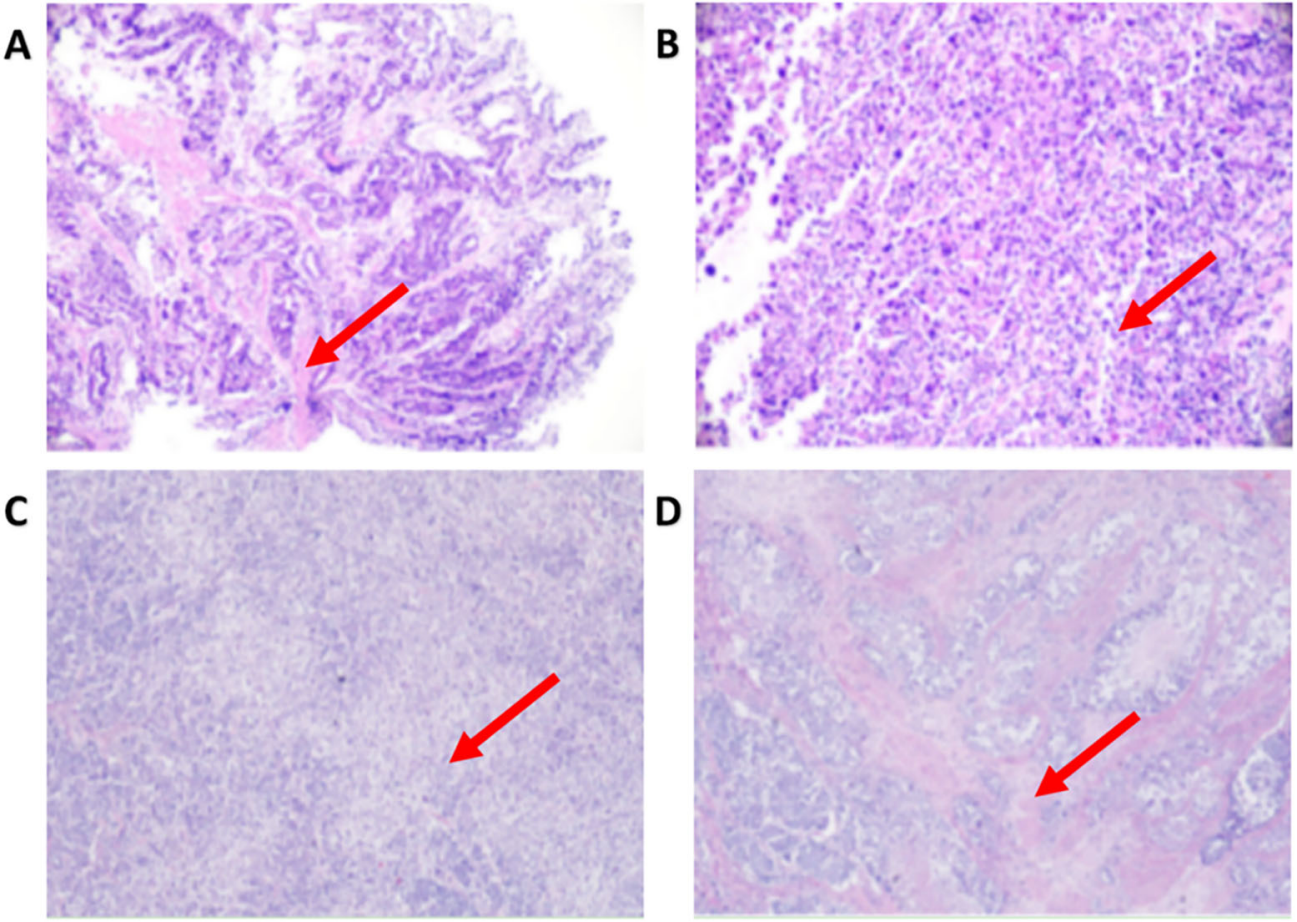
Gastroscopy and histopathological findings of gastric cancer. **(A)** Papillary architecture with tumor cells arranged in branching papillae and fibrovascular cores; **(B)** Diffuse and solid growth pattern with poorly differentiated tumor cells; Pathological evaluation following radical gastrectomy for gastric cancer. **(C)** Postoperative pathology showing diffuse and solid proliferation of neoplastic cells, disordered cellular arrangement, and low differentiation; **(D)** Glandular structures with focal myxoid differentiation.

On March 24, 2023, the patient was admitted to the Second Affiliated Hospital of Shandong First Medical University due to intermittent upper abdominal discomfort for more than 3 months. Contrast-enhanced abdominal CT showed a space-occupying lesion on the cardia and lesser curvature of the stomach, with abnormally enhanced lymph nodes in the hepatogastric space.) (**Figure 1A**). Subsequent gastroscopy revealed an ulcerative mass involving the cardia and lesser curvature of the stomach. Pathological biopsy confirmed the diagnosis of cardio-body adenocarcinoma with a clinical stage of cT4N+M0 (stage III) (**Figures 2A, B**). After a comprehensive evaluation by the multidisciplinary team (MDT), the patient indicated surgery. On April 3, 2023, a total gastrectomy, Roux-en-Y esophagojejunostomy, abdominal lymph node dissection, and omentectomy were performed. Postoperative pathology suggested pT4aN3aM0 (stage IIIB) and confirmed the presence of abdominal lymph-node metastasis (**Figures 2C, D**). After surgery, the patient received two cycles of adjuvant therapy: the first cycle was started on May 10, 2023, and the SOX regimen was used as adjuvant chemotherapy, with oxaliplatin 180 mg (D1) and S-1–40 mg (d1-14). The second cycle of treatment was included in the clinical trial “camrelizumab combined with SOX regimen in the adjuvant treatment of resectable advanced gastric cancer or gastroesophageal junction adenocarcinoma” conducted by our department (Chinese Clinical Trial Registry number: ChiCTR2100051682, International Registry number: NCT05184946), who received camrelizumab 200 mg (D0) combined with SOX regimen on June 4, 2023. Liver function was normal before the second cycle of treatment, but jaundice symptoms developed approximately 20 days after the end of treatment.

### Past medical, social, and family history

The patient was a 76-year-old man with gastric cancer who underwent radical gastrectomy in April 2023; the postoperative course was uncomplicated. He denied alcohol misuse, smoking, or illicit drug use. Psychosocial history was unremarkable, with adequate family support and no identified barriers to care or adherence. Epidemiological history was negative for viral hepatitis risk factors, including schistosomiasis, blood product exposure, and consumption of potentially contaminated food. Family history was noncontributory, with no evidence of viral hepatitis, tuberculosis, hereditary liver disease, or other relevant infectious or genetic disorders among first-degree relatives.

### Physical examination

Vital signs were stable (BP 98/62 mmHg, HR 81 bpm, RR 20/min, T 36.6 °C). The patient was alert and oriented, with no evidence of hepatic encephalopathy (asterixis negative). Marked scleral and cutaneous jaundice was present. There were no stigmata of chronic liver disease (e.g., palmar erythema or spider angiomas). The abdomen was soft and non-tender, with no hepatosplenomegaly; shifting dullness was negative. No ascites or lower-extremity edema was noted.

### Laboratory tests

Total bilirubin (TBIL): peak level 411.9 µmol/L (reference range: 2–25); direct bilirubin (DBIL): peak level 390.4 µmol/L (reference range: 1.7–8); indirect bilirubin (IBIL): peak level 158.3 µmol/L (reference range: 2–14); alanine aminotransferase (ALT): 200 U/L (reference range: 9–60); aspartate aminotransferase (AST): 257.0 U/L (reference range: 15–40); alkaline phosphatase (ALP): 554.0 U/L (reference range: 42–125); gamma-glutamyl transferase (GGT): 366.0 U/L (reference range: 10–60); prothrombin time activity (PTA): 30.4%; international normalized ratio (INR): 2.28. Serological tests for viral hepatitis A–E and autoimmune liver disease were negative. Anti-ENA antibody panel was negative. ANA antibody profile: negative. Serum immunoglobulin G (IgG) levels were within the normal range. Six Epstein-Barr virus antibodies: negative. Cytomegalovirus (CMV) IgG: negative. Tumor markers: carcinoembryonic antigen (CEA): 5.00 ng/mL (reference range: 0–5); alpha-fetoprotein (AFP): 2.201 ng/mL (reference range: 0–10). Based on the peak ALT and ALP values at admission relative to the upper limit of normal (ULN), the R value was calculated as 0.75. This score indicates a predominant cholestatic injury pattern (R ≤ 2), consistent with the clinical presentation; the patient was diagnosed with ICI-related Grade 4 IMH.

### Imaging examination

**Figure 3:**
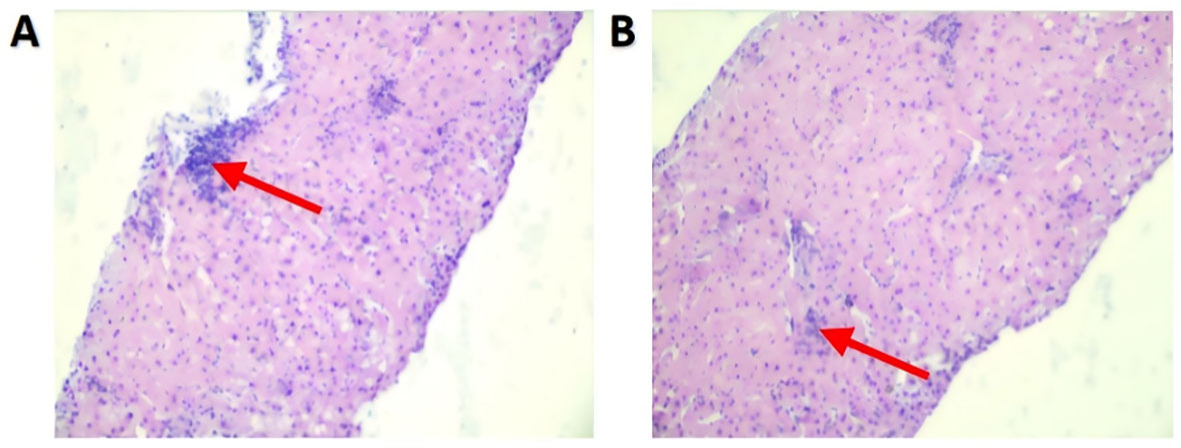
**(A, B)** Liver biopsy showing well-preserved hepatic architecture with regularly arranged cords. Mild hepatocellular edema is present, accompanied by focal bile stasis. Lymphocytic aggregates are observed within the portal tracts, indicating a localized inflammatory.

Abdominal CT showed postoperative changes in the gastric mass with no significant difference compared to the preoperative scan (**Figure 1B**). Liver morphology and density appeared normal. Color Doppler ultrasound of the gastrointestinal system revealed that the middle and lower segments of the common bile duct were not clearly visualized, suggestive of cholestasis. There was no dilatation of the portal vein or intrahepatic and extrahepatic bile ducts. Ultrasound-guided liver biopsy demonstrated preserved hepatocyte alignment, mild hepatocellular edema, focal cholestasis, and lymphocytic aggregation in the portal tracts, findings consistent with possible immune-mediated hepatitis (**Figures 3A, B**).

### Diagnosis

Subacute liver failure (SALF); Immune-mediated hepatotoxicity (IMH) (CTCAE v5.0 grade 4); Postoperative poorly differentiated gastric adenocarcinoma (pT4aN3aM0, stage IIIB).

### Process of treatment

**Figure 4:**
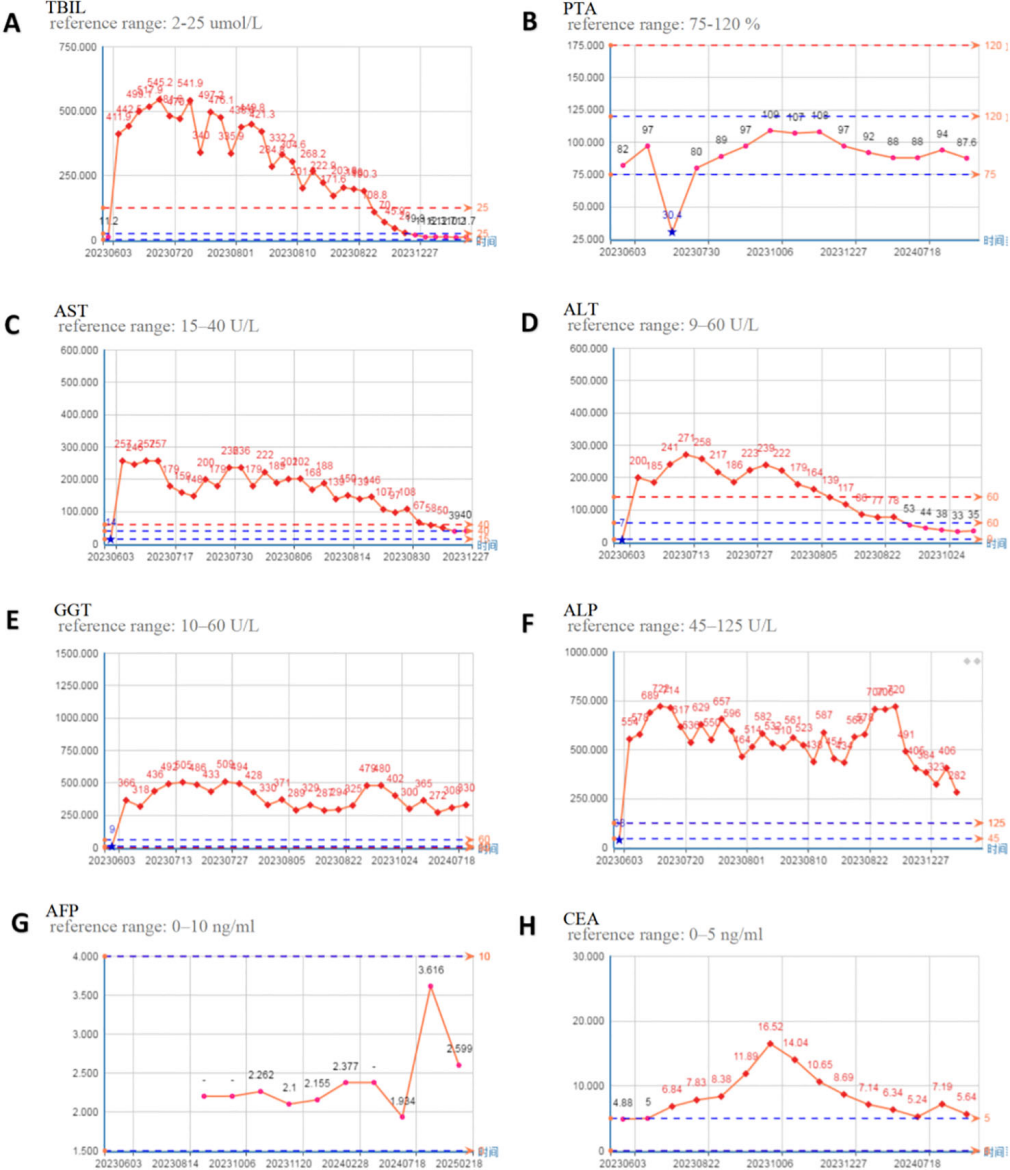
Trends in liver function-related indicators and tumor markers since June 3, 2023. **(A)** TBIL; **(B)** PTA; **(C)** AST; **(D)** ALT; **(E)** GGT; **(F)** ALP; **(G)** AFP; **(H)** CEA.

Upon admission, camrelizumab, oxaliplatin, and S-1 were discontinued. Supportive hepatoprotective therapy was initiated, including intravenous magnesium isoglycyrrhizinate (200 mg qd), polyene phosphatidylcholine (465 mg qd), and ademetionine (1 g qd), together with oral ursodeoxycholic acid (250 mg tid). Given the suspected immune-mediated mechanism, intravenous methylprednisolone (100 mg qd) was started; after 5 days, the dose was tapered to 20 mg using a 7-day step-down regimen. Because biochemical improvement remained insufficient, mycophenolate mofetil (750 mg bid) was added on day 4 of corticosteroid therapy. Despite these measures, liver function continued to worsen. After informed consent was obtained from the patient’s family, artificial liver support therapy was initiated from July 28 to August 15, 2023, consisting of alternating PE and DPMAS. In total, two PE sessions (exchange volumes 1,000 mL and 1,150 mL) and three DPMAS sessions were performed (July 28, July 31, August 6, August 10, and August 15). The maintenance medication regimen during the artificial liver treatment included intravenous magnesium isoglycyrrhizinate 200 mg qd, polyene phosphatidylcholine 465 mg qd, methylprednisolone 10 mg qd, and oral mycophenolate mofetil capsule 750 mg bid. Prothrombin activity (PTA) increased significantly from 30.4% to 80% after the first artificial liver treatment. TBIL decreased to 190.3 µmol/L after 2 weeks of treatment, and further decreased to 19.8 µmol/L after 4 months of treatment. From August 16 to 23, 2023, the liver protection regimen was adjusted to intravenous magnesium isoglycyrrhizinate 100 mg qd, polyene phosphatidylcholine 465 mg qd, and adenosylmethionine butydisulfonate 1g qd. Mycophenolate mofetil capsule 500 mg bid, ursodeoxycholic acid 250mg tid, and prednisone tablets 10mg qd were given orally. From August 31 to December 27, he was given oral diammonium glycyrrhizinate capsule 150 mg tid. Treatment of liver function index in the process of continuous improvement, and artificial liver intervention after 4 months, major liver function parameters restored to the normal range: 19.8 (including TBIL mol/L, PTA 97%, 39 U/L AST, the ALT 35 U/L (**Figures 4A–D**).

### Outcomes and follow-up

Under multimodal management with hepatoprotective agents, glucocorticoids, immunosuppressants, and an artificial liver support system, the patient’s liver function improved progressively. Serial measurements showed sustained declines in TBIL, ALT, AST, GGT, and ALP, while PTA gradually recovered to the normal range (**Figures 4A–F**). Reexamination of blood biochemical indicators on August 18, 2023, showed: ALT decreased from 239 U/L to 86 U/L, AST decreased from 236 U/L to 107 U/L, DBIL decreased from 390.4 µmol/L to 160.7 µmol/L, and ALP decreased from 657 U/L to 565 U/L, suggesting that liver function was significantly improved. On December 27, 2023 (4 months after discharge), the reexamination showed that the liver function indexes returned to the normal range. Since then, imaging evaluation has shown no recurrence and stable disease (**Figures 1C, D**), and the levels of tumor markers have not increased significantly (**Figures 4G, H**). According to CSCO Guidelines for the diagnosis and treatment of Antineoplastic Drug-Associated Liver Injury (**Supplementary Figure 1**), this case involved CTCAE v5.0 Grade 4 IMH complicated by subacute liver failure, therefore ICIs and oxaliplatin were permanently discontinued, and S-1 monotherapy was completed for 1 year. Up to the last follow-up in August 2025, the patient’s liver function remained normal, and no tumor recurrence was observed (**Supplementary Figure 2**).

## Discussion

With the expanding clinical application of ICIs, irAEs have become an increasingly important concern. IMH is a clinically significant hepatic irAE, with a reported incidence of approximately 1%–15% (12, 13). Evidence suggests that ICI therapy increases the overall risk of liver injury (relative risk (RR) = 1.40), particularly severe events (RR = 2.55) (14). A meta-analysis of 613 irAE-related deaths revealed that 124 cases (20.2%) were attributed to IMH (15), and an international multicenter melanoma analysis further indicated that IMH carried one of the highest mortality rates among irAEs (15). Against this background, the present case illustrates an unusually rapid-onset, high-grade IMH complicated by subacute liver failure (SALF), underscoring the need for timely recognition and escalation of care.

IMH remains a diagnosis of exclusion and requires careful differentiation from autoimmune hepatitis (AIH) and other causes of acute liver dysfunction. Classically, AIH is characterized by hypergammaglobulinemia (typically elevated IgG), positive autoimmune serologies, and histologic interface hepatitis with a prominent plasma-cell component (16). In our patient, serum IgG was within the normal range, and autoimmune serologies were negative (including ANA and anti-ENA), making AIH less likely. According to European Society for Medical Oncology (ESMO) guidance, liver biopsy is not routinely required when the clinical course after ICI exposure is typical and alternative etiologies have been excluded; however, histopathological assessment is recommended for atypical presentations or inadequate response to initial therapy (7, 17). In this case, biopsy was pursued because etiological attribution was complicated by combination therapy and the patient rapidly progressed to SALF, prior to escalation to intensive immunosuppression and extracorporeal support. Histopathology supported an immune-mediated phenotype and increased confidence in the working diagnosis and helped deprioritize competing etiologies. In addition, applying the R-value framework recommended in the Chinese Guidelines for drug-induced liver injury (DILI) (6, 18), the R value at onset was 0.75 (ALT 200 U/L; ALP 554 U/L), indicating a cholestatic pattern consistent with the disproportionate elevation of alkaline phosphatase and bilirubin.

IMH most commonly occurs 4–12 weeks after ICI initiation or after 1–3 doses (19), whereas our patient developed severe liver injury approximately 20 days after starting camrelizumab and progressed rapidly to SALF, representing an uncommon, fulminant clinical course. A structured work-up was performed to exclude alternative etiologies. Infectious causes were ruled out by negative serologic testing for hepatitis viruses (A–E), cytomegalovirus (CMV), and Epstein–Barr virus (EBV). Mechanical obstruction and vascular causes were also excluded: despite the cholestatic presentation, abdominal CT and color Doppler ultrasound showed no intrahepatic or extrahepatic bile duct dilatation and demonstrated normal portal venous flow. The temporal relationship and clinicopathologic correlation were more consistent with an immune-mediated process than with chemotherapy-related hepatotoxicity. Liver function remained stable during the initial SOX regimen but deteriorated rapidly after camrelizumab was introduced. Together with biopsy findings of portal lymphocytic aggregates, this pattern supports IMH as the most likely driver of SALF in this patient.

From a mechanistic perspective, irAEs are complex and likely multifactorial. In the liver, PD-1/PD-L1 signaling contributes to immune tolerance by restraining excessive intrahepatic T-cell activation; blockade of this pathway may lower the threshold for immune-mediated hepatocyte and/or bile duct injury (20, 21). Other proposed contributors include off-target immune activation and antibody-mediated tissue injury, although their relative roles likely vary across patients. Additionally, non–mutually exclusive mechanisms have been proposed, including antigen cross-reactivity/epitope spreading and cytokine-associated amplification of inflammation (22). In our patient, the short latency (~20 days after exposure) and IMH CTCAE v5.0 grade 4 severity are compatible with a rapid, clinically meaningful disruption of hepatic immune tolerance after PD-1 pathway blockade; however, mechanistic interpretation remains hypothesis-generating in a single-case report, particularly in the absence of longitudinal immune profiling.

Therapeutically, the patient showed limited biochemical response to first- and second-line hepatoprotective therapy. When total bilirubin increased to 171 μmol/L, artificial liver support was initiated in parallel with continued hepatoprotective agents, corticosteroids with adjunct immunosuppression, and supportive care. Following this multimodal escalation, hepatic synthetic function improved substantially (prothrombin activity (PTA) 30.4% → 80% → 97%), which temporally coincided with clinical stabilization and subsequent recovery. Prior reports suggest that PE can remove circulating bilirubin-bound toxins and inflammatory mediators and has been associated with immunologic shifts (e.g., changes in regulatory T-cell/Th17-related indices) (23); however, patient-level immune measurements were not available in our case, and such effects should be interpreted as literature-based hypotheses. The 2023 guideline for artificial liver blood purification systems emphasizes the potential value of timely initiation in appropriate candidates (24). DPMAS has also been used for bilirubin clearance and reduction of selected inflammatory mediators in cholestatic injury (25). In this case, a sequential PE–DPMAS strategy was adopted with the intent to support detoxification while reducing overall plasma utilization.

For glucocorticoid-refractory severe IMH, escalation to a multimodal strategy integrating immunosuppressants and artificial liver support may be considered in selected patients as a bridge to hepatic recovery. In rapidly progressive cases, such an approach may help stabilize liver function while definitive recovery evolves, although the effectiveness of individual components cannot be determined from a single case.

This case has several notable strengths. We performed a structured etiological work-up to exclude competing causes, confirmed IMH histologically in a complex post-chemotherapy context, and provided detailed documentation of a multimodal rescue approach for subacute liver failure. Several limitations should also be noted. As a single-case report, the observations have limited generalizability. Although the temporal relationship and clinicopathologic correlation favored IMH, concomitant oxaliplatin and S-1 exposure remains a potential confounder. Because multiple interventions were initiated in parallel, the relative contribution of each modality cannot be determined. Finally, the absence of longitudinal immune profiling limits mechanistic insight.

Despite these limitations, this case supports considering closer liver function monitoring in selected high-risk patients (e.g., advanced age or pre-existing hepatic burden). In addition to baseline testing, repeat liver biochemistry and coagulation assessment early after treatment initiation and before each cycle may be reasonable. Early use of hepatoprotective agents when transaminases begin to rise may also be considered, although the optimal indications and protocols require further study. Future work should address (1) criteria and protocols for hepatoprotection in high-risk groups; (2) early biomarkers and timing of corticosteroid intervention; and (3) initiation thresholds and therapeutic windows for artificial liver support in immune-related acute/subacute liver failure.

## Conclusions

In summary, we report a patient with camrelizumab-associated IMH (CTCAE v5.0 grade 4) complicated by subacute liver failure. The patient improved after a multimodal approach including hepatoprotective therapy, corticosteroids with adjunct immunosuppression, and sequential artificial liver support using PE and DPMAS, with subsequent normalization of liver biochemistry on follow-up. Given the single-case nature of this report and the absence of longitudinal immune profiling, mechanistic inferences—particularly regarding immune modulation—should be interpreted cautiously. These observations are hypothesis-generating and suggest that artificial liver support may be considered as an adjunct in refractory severe IMH, warranting evaluation in larger prospective cohorts.

## Data Availability

The original contributions presented in the study are included in the article/**Supplementary Material**. Further inquiries can be directed to the corresponding author.

## Supplementary material

Supplementary Figure 1 Treatment algorithm for IMH following ICI therapy according to CSCO guidelines. IMH, immune-mediated hepatitis; ICI, immune checkpoint inhibitor; MMF, mycophenolate mofetil; UDCA, ursodeoxycholic acid; ATG, antithymocyte globulin; IVIG, intravenous immunoglobulin.

Supplementary Figure 2 Timeline of diagnostics, therapeutics, and outcomes. The clinical course is illustrated from March 2023 to August 2025. Following radical gastrectomy and adjuvant chemotherapy (SOX plus camrelizumab), the patient developed Grade 4 immune-mediated hepatotoxicity (IMH) progressing to subacute liver failure. On July 5, 2023, diagnosis was confirmed (R value = 0.75; liver biopsy), and camrelizumab/SOX were permanently discontinued. Multimodal management involved stepwise immunosuppression (methylprednisolone and mycophenolate mofetil) and an artificial liver support regimen comprising three sessions of double plasma molecular adsorption system (DPMAS) alternating with two sessions of plasma exchange (PE) from July 28 to August 15. Follow-up demonstrated complete normalization of liver function (TBIL 19.8 µmol/L; PTA 97%) by December 27, 2023. ICI, immune checkpoint inhibitor; IMH, immune-mediated hepatotoxicity; SOX, S-1 and oxaliplatin; DPMAS, double plasma molecular adsorption system; PE, plasma exchange; TBIL, total bilirubin; INR, international normalized ratio; PTA, prothrombin activity; ALT, alanine aminotransferase.
