# Supplementary figures and images for: Subacute liver failure caused by immunotherapy in a patient with gastric cancer was improved after multimodal treatment with artificial liver combined with liver-protective drugs: a case report
Source: Front Immunol. 2026 Feb 13;17:1755661. doi: 10.3389/fimmu.2026.1755661 (PMC12946037; doi:10.3389/fimmu.2026.1755661)

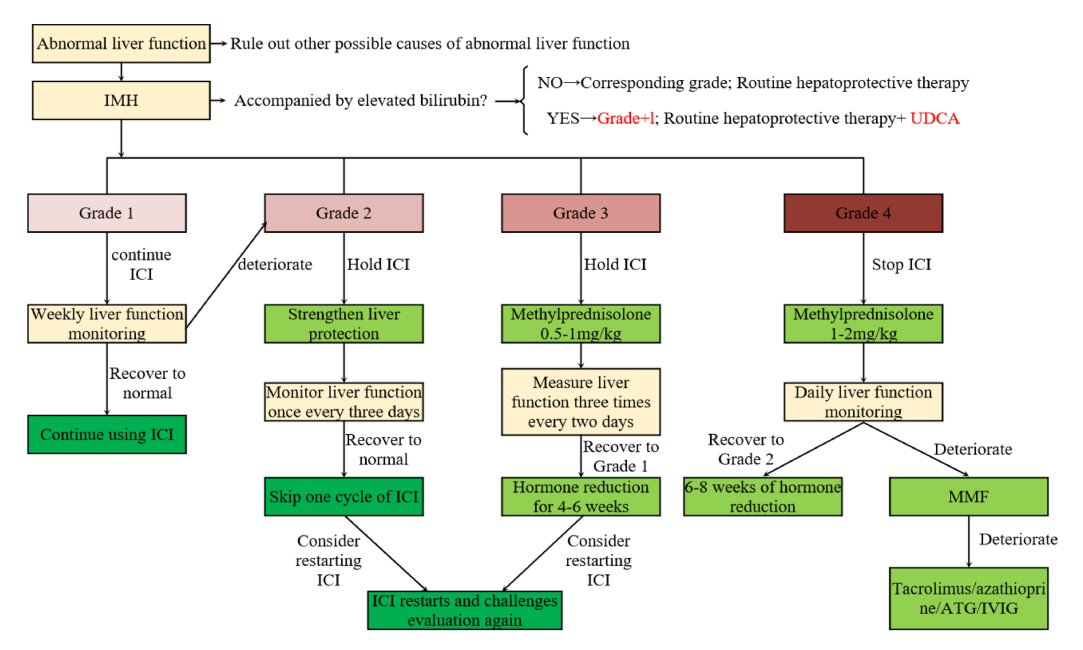

Supplement: Supplementary Figure 1 — Treatment algorithm for IMH following ICI therapy according to CSCO guidelines. IMH, immune-mediated hepatitis; ICI, immune checkpoint inhibitor; MMF, mycophenolate mofetil; UDCA, ursodeoxycholic acid; ATG, antithymocyte globulin; IVIG, intravenous immunoglobulin. [file Image1.tif]

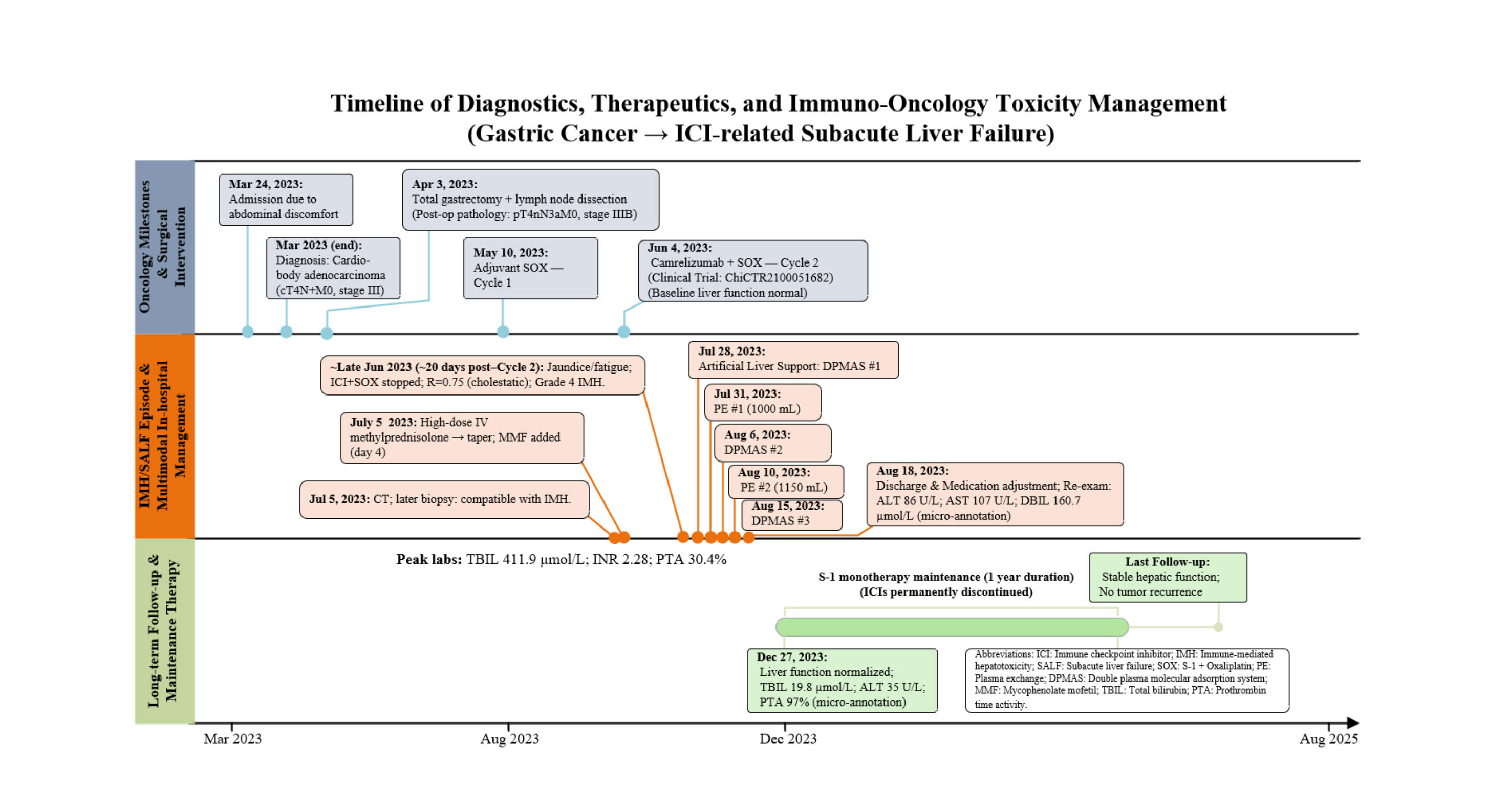

Supplement: Supplementary Figure 2 — Timeline of diagnostics, therapeutics, and outcomes. The clinical course is illustrated from March 2023 to August 2025. Following radical gastrectomy and adjuvant chemotherapy (SOX plus camrelizumab), the patient developed Grade 4 immune-mediated hepatotoxicity (IMH) progressing to subacute liver failure. On July 5, 2023, diagnosis was confirmed (R value = 0.75; liver biopsy), and camrelizumab/SOX were permanently discontinued. Multimodal management involved stepwise immunosuppression (methylprednisolone and mycophenolate mofetil) and an artificial liver support regimen comprising three sessions of double plasma molecular adsorption system (DPMAS) alternating with two sessions of plasma exchange (PE) from July 28 to August 15. Follow-up demonstrated complete normalization of liver function (TBIL 19.8 µmol/L; PTA 97%) by December 27, 2023. ICI, immune checkpoint inhibitor; IMH, immune-mediated hepatotoxicity; SOX, S-1 and oxaliplatin; DPMAS, double plasma molecular adsorption system; PE, plasma exchange; TBIL, total bilirubin; INR, international normalized ratio; PTA, prothrombin activity; ALT, alanine aminotransferase. [file Image2.tif]
